# A 17.1 kb duplication downstream GATA6 is strongly associated with egg weight in chicken

**DOI:** 10.1186/s12864-025-11888-0

**Published:** 2025-08-20

**Authors:** Lei Wang, Shaobo Han, Weijian Fan, Yangming Pan, Bo Yuan, Guanyu Hou, ShiJun Li

**Affiliations:** 1 Key Laboratory of Agricultural Animal Genetics, Breeding and Reproduction, Ministry of Education, Huazhong Agricultural University, Wuhan, Hubei Province 430070 China; 2 Yazhouwan National Laboratory, Sanya, 572024 P. R. China; 3 Key Laboratory of Smart Farming for Agricultural Animals, Ministry of Education, Huazhong Agricultural University, Wuhan, Hubei Province 430070 China; 4 Hubei Hongshan Laboratory, Wuhan, Hubei Province 430070 China; 5 Chinese Academy of Tropical Agricultural Sciences, Haikou, Hainan Province 570100 China

**Keywords:** Egg weight, Danzhou chicken, Duplication, GATA6

## Abstract

Egg weight (EW) is one of the most economically important traits in chickens. Recent statistics show that the EWs of most yellow-feathered chickens in China fall within the 40 ~ 50 g range, while, Danzhou (DZH) chickens exhibit significantly lower EW compared to this range. Here, we aim to identify candidate genes and regulatory variants involved in EW control by conducting a genome-wide association study (GWAS) with high-density SNPs obtained from whole-genome sequencing (WGS). We performed a GWAS using WGS data from 76 DZH chickens and 120 Wenchang (WCH) chickens to identify loci associated with the unique EW phenotype in DZH chickens. Using a mixed linear model, we identified an association signal at 102,727,028 bp ~ 102,822,629 bp on chromosome 2. This signal is adjacent to the GATA6, which plays a key role in liver development. Given the liver’s essential role in synthesizing yolk precursors, we hypothesized this signal region might influence EW through liver function. Although SNPs and indels within this signal region were ruled out as causal variants for EW, SV-gwas between DZH and WCH revealed a 17.1 kb duplication (dup, chr2:102,721,722–102,738,778 bp) approximately 66.6 kb downstream of GATA6. In the F_2_ population of 118 individuals derived from DZH and WCH chickens, PCR and qPCR of the dup revealed significant differences in EW. Both heterozygous (dup/+) and homozygous (dup/dup) individuals showed significantly higher minimum and average EW compared to wild-type (+/+) controls. Moreover, the maximum EW observed in homozygotes was also significantly elevated relative to the wild-type. Dual-luciferase assays confirmed the presence of an open chromatin region within the dup, validated by ATAC-seq, which exhibited enhanced regulatory activity. Additionally, GATA6 expression levels measured by qPCR were significantly higher in high EW individuals with heterozygous or homozygous genotypes compared to wild-type individuals with low EW. In conclusion, our findings establish, for the first time, that a 245 bp enhancer-containing dup in GATA6’s regulatory region critically regulates EW variation in yellow-feathered chickens, providing a potential molecular marker for breeding programs targeting egg size optimization.

**Supplementary Information:**

The online version contains supplementary material available at 10.1186/s12864-025-11888-0.

## Introduction

Egg weight (EW) is one of the most important economic traits in the poultry industry [1]. EW exhibits strong correlations with quantitative traits like body weight and body size [2]. Controlling EW is therefore crucial for poultry breeding. Most Chinese yellow-feathered chicken breeds exhibit EW values ranging from 40 to 50 g. These breeds include Wenchang chicken (WCH), Huanglang chicken (HL), Huaibei Mottled chicken (HBM), Ningdu Yellow chicken (NDY), Wuhua chicken (WH), Zhengyang Three-Yellow chicken (ZYTY), Yunyang Large chicken (YYL), and Shennongjia Large chicken (SNJL). In contrast, Danzhou chicken (DZH) displays a remarkably lower average EW of 35.05 ± 2.40 g compared to other yellow-feathered breeds. This unique characteristic makes DZH chicken a valuable resource population for studying the genetic mechanisms underlying EW. This unique phenotype positions DZH chicken as a valuable model for investigating the genetic basis of EW regulation. Research has consistently shown that genetic factors are predominant in determining EW [3]. Identifying the key genes or regulatory loci associated with EW can significantly accelerate the breeding process and bring long-term benefits to poultry breeding.

To date, the genetic basis of EW has been extensively studied in poultry. According to the animal QTL database, a total of 248 QTLs associated with EW have been identified, spanning 18 autosomes and the Z chromosome [4]. Recently, genotyping array data studies have also identified QTLs related to EW in yellow-feathered chickens on chromosomes 2, 4, 5, 6, 8, 20, 23, 28, and Z [5, 6]. One of the critical regulatory systems governing egg yolk precursor synthesis and follicular development is the liver-blood-ovary signaling axis. Within this axis, the very low-density lipoprotein receptor (VLDLR) and cathepsin D (CTSD) play crucial roles in the yolk deposition process by transporting liver-derived yolk precursor materials [7, 8]. Notably, polymorphisms in the CTSD (2614T > C and 5274G > T) are closely associated with both yolk weight (YW) and EW [9]. In zebrafish, retinol-binding protein 4 (Rbp4), a gene essential for liver development and function, has been demonstrated to play a vital role in transporting and utilizing yolk precursors [10]. However, while genotyping array data have proven valuable in identifying QTLs associated with EW, they are limited in resolving the structural variants (SVs) and regulatory mechanisms underlying these QTL regions [11]. This limitation is significant because SVs often play key roles in gene regulation and can substantially influence complex traits like EW. Unlike genotyping array data, whole-genome sequencing (WGS) data offers a more comprehensive approach, allowing for finer-scale mapping and the SVs identification [12]. WGS data provides the resolution necessary to pinpoint causative mutations within QTL regions [13]. While numerous QTLs associated with EW have been identified in yellow-feathered chickens, the underlying genetic mechanisms remain elusive.

DZH chickens represent a valuable population for studying EW. To investigate the potential causative mutations regulating EW in DZH chickens, based on the WGS data from the DZH and WCH chicken populations, we used a mixed linear model to identify candidate genomic regions and SVs associated with EW preliminarily. These SVs were further validated in an F_2_ population of DZH and WCH chickens. By integrating multi-omics data and performing dual-luciferase assays, we further investigated the role of these SVs in regulating EW. This provides a robust molecular genetic test for the selection of egg weight in yellow-feathered chickens.

## Methods

### Animal rearing and welfare

At the Chinese Academy of Tropical Agricultural Sciences, we constructed an F_2_ population based on 5 male WCH chickens and 25 female DZH chickens. This initial cross produced 97 F1 offspring. The F1 generation was then randomly interbred to produce an F_2_ population consisting of 264 individuals. In 42 weeks, 118 F_2_ individuals were randomly selected to collect blood from subwing vein after the data of egg number and egg weight phenotype were recorded in 35 days. 120 WCH chickens and 76 DZH chickens were selected for this study. and blood collection. The chickens were housed individually in cages with ad libitum access to feed, and EW data were collected at 42 weeks of age. 20 individuals with extreme egg weight group were selected and euthanized before liver and oviduct ampulla tissues were collected. In particular, 40 mg ketamine was injected into each individual by intramuscular injection. Femoral artery, carotid artery and abdominal aorta of the anesthetized experimental animals were cut, and the heart was pierced to bleed, resulting in acute massive bleeding, shock and death. Minimize animal death suffering. The protocol for this study was reviewed and approved by the Institutional Animal Care and Use Committee (IACUC) of Huazhong Agricultural University (Wuhan, China). All procedures complied with ethical guidelines for animal care, ensuring that the welfare of the chickens was prioritized throughout the study.

### Whole-genome sequencing, reads alignment, and variant calling and annotations

120 WCH and 78 DZH chickens Genomic DNA was extracted from blood samples using the traditional phenol-chloroform method. After quality assessment, the genomic DNA was subjected to whole-genome resequencing (WGS) on the DNBSEQ T7 sequencer (BGI, China) with a read length of 150 bp and paired-end (PE) reads. WGS data from 22 Red gallus (RJ) and 66 yellow-feathered chickens (8 HL, 10 HBM, 10 NDY, 10 WH, 8 ZYTY, 10 YYL, and 10 SNJL) were downloaded from the SRA database (Supplementary Table S1). High-quality reads were aligned to the chicken reference genome (GCF_000002315.5) using BWA-MEM (v0.7.12) [14], and short variant calling (SNPs and indels) was performed using the HaplotypeCaller module in GATK (v3.6) [15]. The gvcf files were combined using CombineGVCFs and GenotypeGVCFs [16]. Low-quality variants were filtered out using the VariantFiltration tool [17] with the parameters “QD < 2.0, QUAL < 30.0, MQ < 40.0,” resulting in 24,434,301 high-quality short variants (21,471,712 SNPs and 2,962,589 INDELs). Lumpy (v0.3.1) was used to detect structural variation (SV) on autosomes and perform genotyping, yielding 15,429 high-quality SVs on chromosome 2 [18].

After filtering out variants with minor allele frequency (MAF) < 0.01 and missing genotype > 10% using VCFtools [19], the final variant dataset was obtained. Biallelic 10,042,447 SNPs and 5,650 SVs were extracted for subsequent GWAS analysis. Additionally, SnpEff (v4.1) was used to annotate SNPs and indels, assessing the putatively functional impact of variants on protein-coding genes [20].

### Genome-wide association analysis and linkage disequilibrium analysis

Although there is relatedness among WCH and DZH chickens, the principal component analysis (PCA) was performed to estimate the extent of population structure using GCTA [21]. Subsequently, a genome-wide association study (GWAS) was conducted using a mixed linear model implemented in GEMMA (v0.98.3) [22], with the first four PCAs and body weight as model covariates, that is, fixed effects. False positives were reduced by correcting for kinship. The model details are as follows:

$$\:y=Xb+Zu+e$$

Where y represents the vector of phenotypic values, b is the vector of fixed effects, u is the vector of additive genetic effects, and X and Z are the incidence matrices for b and u, respectively, with e representing the residual effects.

Significant short variant genotype data within the association signals were extracted using VCFtools (v0.1.16). Linkage disequilibrium (LD) analysis was performed using the LDheatmap (v1.0.5) R package [23], and Manhattan plots and LD heatmaps were generated using custom R scripts. Additionally, the alignment results of the BMA format were visualized with the Integrative Genomics Viewer (IGV) to identify the significant SV loci [24].

### PCR and Sanger sequencing of duplication within the candidate region

To validate the 17.1 kb duplication identified in the association region using WGS data, we performed PCR amplification to verify the presence of the duplication using three primers (Supplementary Fig. 1; F1: 5’-ATCTCCAAAATCATGCCACT-3’, F2: 5’- GTTGCTGCTTCTCTGCCTTC-3’, R: 5’-GCAACGGCAACCAAATTATC-3’). The F2 and R primers, which spanned the breakpoint junction of the duplication, amplified a 267 bp fragment when the 17,100 bp tandem duplication was present. Conversely, the F1 and R primers spanning a sequence in dup duplication amplify an 184 bp fragment when the DNA is normal. The PCR reaction mixture (10 µL) included 6µL 2×GS Taq PCR Mix, 4.05µL RNase-free ddH_2_O, 0.15µL each primer (F1, F2 and R), and 1.5µL genomic DNA. The PCR conditions were as follows: initial denaturation at 95 °C for 5 min; 30 cycles of 95 °C for 30 s, 58 °C for 30 s, and 72 °C for 30 s; and a final extension at 72 °C for 5 min and 25 °C for 1 min. PCR products were separated by 3.8% agarose gel electrophoresis. We validated the presence or absence of duplication in a total of 675 samples (80 DZH, 192 WCH, 60 LLB, 31 YYL, 93 SNJL, and 219 F_2_ population).

### Detection of duplication genotypes based on qPCR

To distinguish between heterozygous (dup/+) and homozygous (dup/dup) mutants with duplication, we performed PCR amplification to quantify the dup copy number using four primers (F69: 5’-CTGATAGACCATCCTCCTCC-3’, R347: 5’-GCAACTGAAGGAATTACCCC-3’, F28: 5’-CTGTTCATTGCTGCTCCGTT-3’, R310: 5’-TCTGGATTTTTCGGCGTTCT-3’). The PCR reaction mixture (10 µL) included 5µL 2×GS Taq PCR Mix, 3.8µL RNase-free ddH2O, 0.1µL each primer, and 1µL genomic DNA. The PCR conditions were as follows: initial denaturation at 95 °C for 5 min; 35 cycles of 95 °C for 30 s, 58 °C for 30 s, and 72 °C for 30 s; and a final extension at 72 °C for 5 min and 25 °C for 1 min. The qPCR conditions were as follows: initial denaturation at 95 °C for 30 s; 40 cycles of 95 °C for 5 s and 58 °C for 30 s; and a final extension at 95 °C for 15 s and 55 °C for 15 s and 95 °C for 5s. We validated the duplication genotype in 118 F_2_ population.

### Candidate genes were screened using RNA-seq

Liver and oviduct ampulla (Supplementary Fig. 2) tissues from three multiple-copy, high EW individuals (EW > 40.2 g) and three single-copy, low EW individuals (EW < 33.5 g) were used for RNA extraction. Total RNA was extracted from the collected tissues using standard methods. After quality assessment, mRNA was subjected to transcriptome sequencing on the Illumina HiSeq 2500 platform (Wuhan Gene Shadow, China) with 150 bp paired-end (PE) reads. High-quality reads were aligned to the chicken reference genome (GCF_000002315.5) using hisat2 [25], followed by gene expression quantification using stringtie [26]. Differentially expressed genes (DEGs) were identified using a ballgown with thresholds set at|logFC| >1 and *P*-value < 0.05 (Supplementary Fig. 3 ~ 6). To identify key genes, we intersect the significantly differentially expressed genes with those within 100 kb upstream and downstream of the associated considerable genome region. The genes in this intersection are the key candidate genes.

### Differential genes near duplication were investigated by qPCR

Liver tissues from 6 single-copy low egg weight (EW < 33.5 g) and 6 multi-copy high egg weight (EW > 40.2 g) F_2_ individuals were selected to investigate the expression levels of genes enriched in the duplication regions. we designed one primer pairs (F: 5’- TGCGGGCTCTACACCAAAAT-3’, R: 5’- GCAGTAGTGTTGTTACCAGACC-3’) and conducted qPCR to verify gene expression. The PCR reaction system contained 10 µL, including 5 µL ChamQ Universal SYBR qPCR Master Mix, 3.8 µL RNase-free ddH2O, 0.1 µL forward primer, 0.1 µL reverse primer, and 1 µL cDNA. The PCR cycling conditions were as follows: 95 °C for 5 min; 40 cycles of 95 °C for 30 s, 60 °C for 30 s and 72 °C for 30 s, followed by 72 °C for 5 min and 25 °C for 1 min. The qPCR cycling conditions were as follows: 95 °C for 30 s; 40 cycles of 95 °C for 5 s and 60 °C for 30 s, followed by 95 °C for 15 s and 65 °C for 15 s.

### ATAC-seq and dual-luciferase screening of variants in regulatory regions

To further explore regulatory activity in dup, we collected Liver tissues from 2 single-copy low egg weights and 2 multi-copy high egg weights for ATAC-seq. Bowtie2 [27] and MASC2 [28] were used for mapping and screening of open chromatin regions (OCR), and the DEseq2 [29] was used to further screening significant differences OCR (Supplementary Fig. 9, *P*-value < 0.05). Two significant differences OCR in duplication were selected as targeting fragments. We designed two primer pairs for PCR amplification. The F_2_ samples were used as templates to construct pGL3-promoter reporter vectors [30]. Two fragments were cloned into the vectors, and the constructs were transfected into DF1 cells to assess the potential regulatory function of the duplication regions using a dual-luciferase reporter assay [31]. All experiments were performed with three biological and three technical replicates. The firefly luciferase activity was normalized using the renal luciferase activity of each sample.

### Statistical and analysis

Statistical analyses were conducted to evaluate significant differences in EW and related the duplication genotypes and gene expression. The ggplot2 [32] and GraphPad Prism [33] were used to analyze the qPCR result and dual-luciferase assay results. An unpaired t-test was applied to determine the significance of differences between groups. All t-tests were two-tailed, with a significance threshold set at *P* < 0.05 to ensure robustness in detecting genuine trait differences.

## Result

### F_2_ population egg weight statistics

**Fig. 1:**
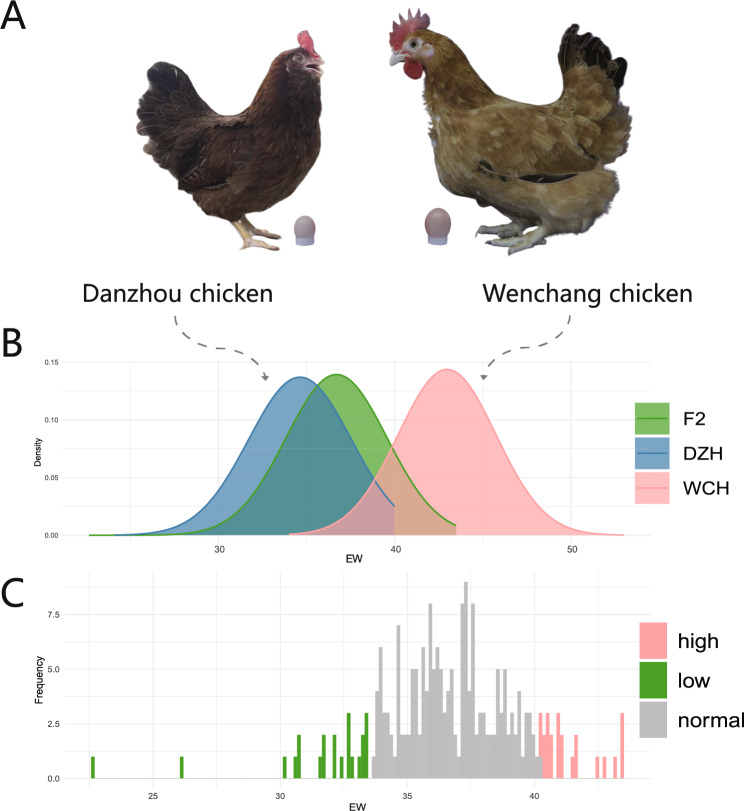
Egg weight statistics for the DZH chicken, WCH chicken and F_2_ population constructed from DZH chicken and WCH chicken. **A** Photos of DZH chickens, WCH chickens, and their eggs; **B** Normal distribution curves of EWs in DZH chickens, WCH chickens and the F_2_ population. The green area represents the F_2_ population, the blue area represents DZH chickens, and the red area represents WCH chickens; **C** EW distribution of the F_2_ population. The red area represents individuals with EW greater than 40.2 g, the green area represents individuals with EW less than 33.5 g, and the gray area represents individuals with EW between 33.5 g and 40.2 g

To investigate the genetic basis of the small egg trait in DZH chickens, we measured egg weight (EW) in DZH chickens, WCH chickens (Fig. 1A), and their F_2_ population. The average EW was 35.05 ± 2.40 g for DZH chickens, 42.97 ± 2.77 g for WCH chickens, and 36.54 ± 2.86 g for the F_2_ population (Fig. 1B). WCH chickens produce 22.6% heavier eggs than DZH chickens. In the F_2_ population, the top 10% of EW had weights exceeding 40.2 g, while the bottom 10% with EWs weighed less than 33.5 g (Fig. 1C). These findings confirm that EW differs significantly between DZH and WCH chickens.

### GWAS identified a 17.1 kb tandem duplication associated with breed Characteristics

**Fig. 2:**
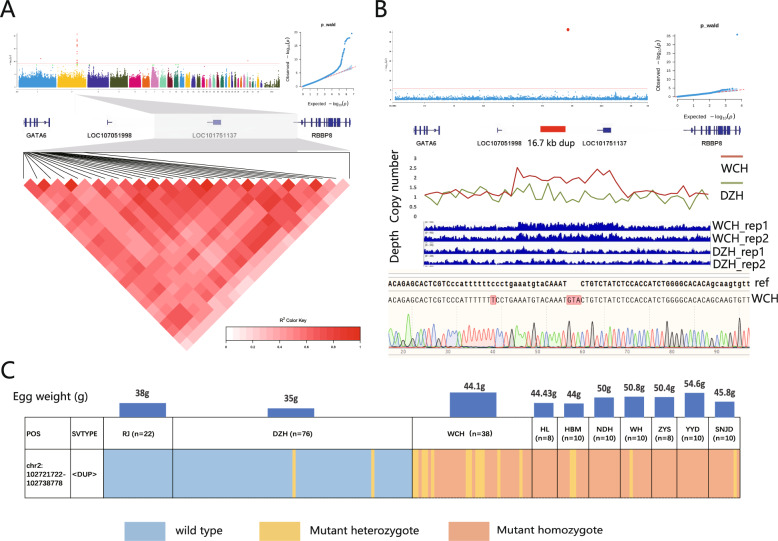
Genetic analysis of EW based on GWAS. **A** Genome-wide association analysis (GWAS) and linkage disequilibrium analysis between DZH and WCH chickens. The red solid line represents the 1% significance threshold after the Bonferroni correction, while the gray dashed line represents the 5% significance threshold after the Bonferroni correction. The gray areas in the gene structure diagram denote QTL regions. **B** SV-GWAS of chromosome 2 and SV Sanger sequencing. In SV-GWAS, the red solid line represents the 1% significance threshold after the Bonferroni correction, while the gray dashed line represents the 5% significance threshold after the Bonferroni correction. Red blocks indicate the position and size of significant SV in the genome. **C** Distribution of SV genotypes on chromosome 2 across 10 chicken breeds. The blue blocks represent the SV wild-type, the yellow blocks represent the SV mutant heterozygous, and the red blocks represent the SV mutant homozygous. RJ represents Red gallus, DZH represents Danzhou chicken, WCH represents Wenchang chicken, HL represents Huanglang chicken, HBM represents Huaibei Mottled chicken, NDY represents Ningdu Yellow chicken, WH represents Wuhua chicken, ZYTY represents Zhengyang Three-Yellow chicken, YYL represents Yunyang Large chicken, and SNJL represents Shennongjia Large chicken

We used WGS data from DZH and WCH chickens to perform a GWAS with a mixed linear model (Bonferroni-corrected *P* < 0.05,−log_10_*P* = 8.04). We identified 25 significant SNPs on chromosome 2 (Supplementary Table S2), spanning positions 102,727,028 bp to 102,822,629 bp, all clustered within a single association peak (Fig. 2A). This genomic annotation region harbors LOC101751137, with three neighboring genes (LOC107051998, GATA6, and RBBP8) located within 100 kb. BLAST analysis showed that there were highly homologous mRNA sequences of GATA6 in LOC101751137 and LOC107051998. These genes are likely candidates for influencing EW.

To further investigate causal mutations affecting EW in DZH chickens, we first annotated significant SNPs and indels within the chromosome 2 QTL but found no functional variants. This allowed us to exclude SNPs and indels in this QTL as functional mutations associated with EW regulation. We then conducted a genome-wide structural variant (SV) calling on WGS data from 202 chickens to identify potential functional variants. Through SV-GWAS, we discovered a significant duplication within the chromosome 2 QTL (Fig. 2B, *P* = 8.18 × 10^−31). Notably, 24 SNPs located on this duplication exhibited moderate linkage disequilibrium (r² >0.6).

PCR amplification (Supplementary Fig. 1) and Sanger sequencing verified that the duplication (dup) was precisely 17.1 kb in length, spanning positions 102,721,722–102,738,778 bp on chromosome 2. A 1 kb sliding window analysis of coverage depth revealed that the WCH chickens were dup homozygous of multiple copy, while DZH chickens exhibited a dup wild-type of single-copy (Fig. 2B). Sanger sequencing confirmed that the dup is 17.1 kb tandem duplication, with a GTA sequence connecting the repeated segments. Given that this dup could be involved in regulating the EW trait in DZH chickens, we performed genotyping on this dup using WGS data from Red gallus (RG) and nine other Chinese yellow-feathered chicken breeds (DZH, WCH, HL, HBM, NDY, WH, ZYTY, YYL and SNJL). The results indicated that in all sampled breeds with an average egg weight above 40 g (WCH, HL, HBM, NDY, WH, ZYTY, YYL and SNJL), the dup was either homozygous or heterozygous for the mutant form. In contrast, among breeds with an average egg weight below 40 g (RG and DZH), all RGs and most DZH chickens were wild-type for the dup allele, except for two Danzhou individuals, which were heterozygous (Fig. 2C). PCR results confirmed the WGS genotyping, providing consistent evidence (Table 1, Supplementary Fig. 12 ~ 14) and suggesting that the dup may affect EW regulation in yellow-feathered chickens.

**Table 1:** PCR validation of duplication genotypes in 5 chicken breeds

	DZH	WCH	LLB	YYL	SNJL
Single dup	71	0	0	0	2
Multiple dup	9	192	59	31	93
*DZH* represents Danzhou chicken, *WCH* represents Wenchang chicken, *LLB* represents Luling black chicken, *YYL* represents Yunyang Large chicken, and *SNJL* represents Shennongjia Large chicken					

### A 17.1 kb tandem duplication was significantly correlated with egg weight in F_2_ population

**Fig. 3:**
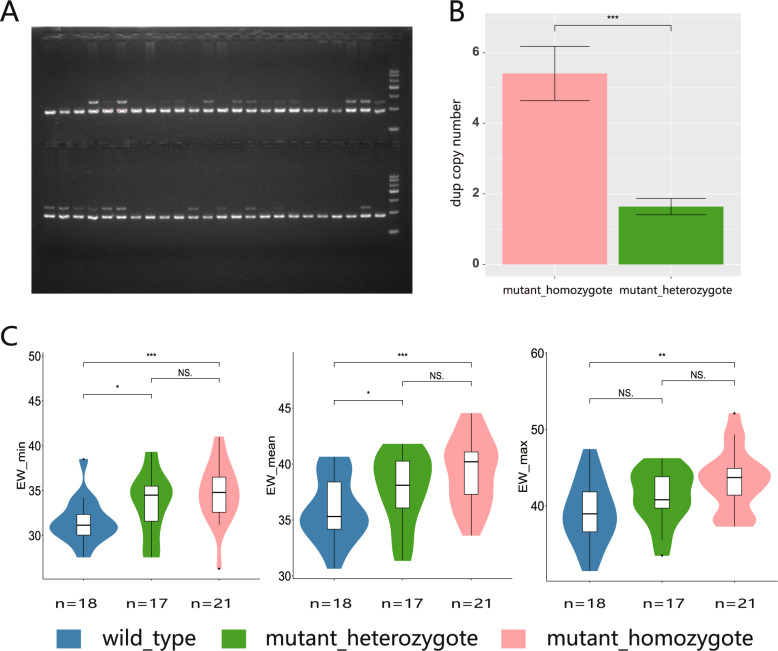
Statistical association analysis of EW and duplication genotype in F_2_ population. **A** PCR results of the duplication in the F_2_ population. **B** qPCR results of the copy number in the F_2_ population. Error line represents standard deviation (SD). **C** Statistical analysis of EW and duplication genotype in F_2_ population. The blue block represents the wild type, the green block represents mutant heterozygotes, and the red block represents mutant homozygotes. * indicating *P* < 0.05 significance, ** indicating *P* < 0.01 significance, and *** indicating *P* < 0.001 significance

To further confirm whether the 17.1 kb tandem duplication (dup) is involved in egg weight regulation, we quantified the copy number of this dup in the 118 F_2_ population using PCR and qPCR of dup (Fig. 3A, Supplementary Fig. 11). Based on gel electrophoresis results, the F_2_ population was categorized into dup wild-type of single copy and dup mutant of multiple copy. qPCR results further distinguished dup heterozygotes, with a copy number of 1.64 ± 0.23, from dup homozygotes, with a copy number of 5.17 ± 0.64. Statistical analysis of EW across dup wild-type (+/+), heterozygote (dup/+), and homozygote (dup/dup) groups revealed that dup homozygotes showed significantly higher minimum, average, and maximum EWs than dup wild-type individuals. Dup heterozygotes also exhibited increased considerably minimum and average EWs relative to the wild-type. These findings suggest a positive genetic correlation between dup copy number and EW, indicating that the dup may affect EW.

### GATA6 was identified as a major candidate gene for egg weight

**Fig. 4:**
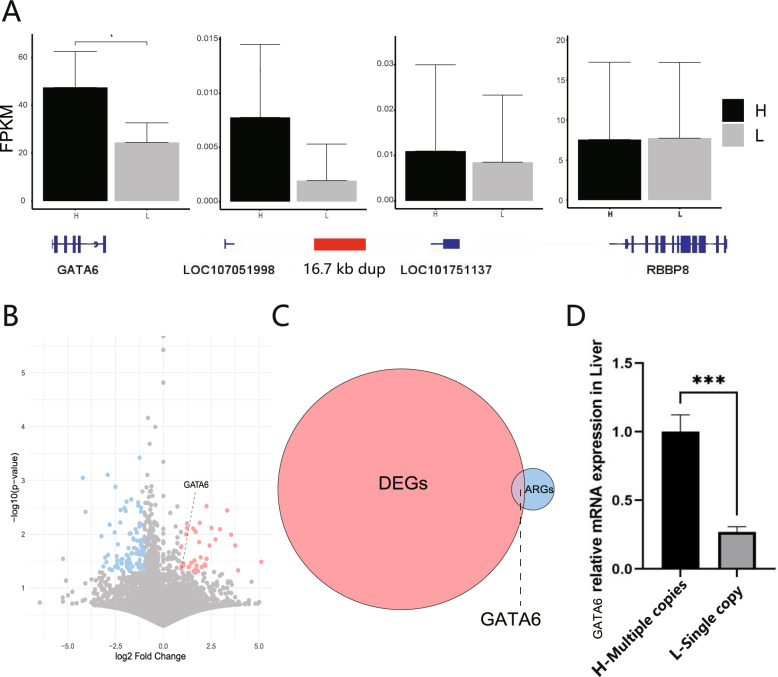
ARGs expression in liver. **A** FPKM of ARGs. **B** DEGs volcano map of liver. **C** Venn diagram of ARGs and DEGs. **D** qPCR results for the GATA6 in liver tissues, with *** indicating a significance level of *P* < 0.001. Error line represents standard deviation (SD)

To identify candidate genes regulating EW, we screened for differentially expressed genes (DEGs) in liver and oviduct ampulla tissues between low EW (dup wild-type, EW < 33.5 g) and high EW groups (dup mutant, EW > 40.2 g). The gene expression data showed that four genes in associated region, only GATA6 was significantly different in the liver (Fig. 4A), but not in the oviduct ampulla (Supplementary Fig. 7). In the liver and the oviduct ampulla, 134 DEGs (Fig. 4A; up: 35, down: 99) and 232 DEGs (Supplementary Fig. 8; up: 51, down: 181) were identified, respectively. However, in Associated region genes (ARGs) and DEGs, only GATA6 gene overlaps (Fig. 4C), so it can be inferred that GATA6 is the candidate gene for EW regulation, consistent with qPCR results (Fig. 4D).

### Luciferase verified the 245 bp Enhancer region in duplication increased gene excpression

**Fig. 5:**
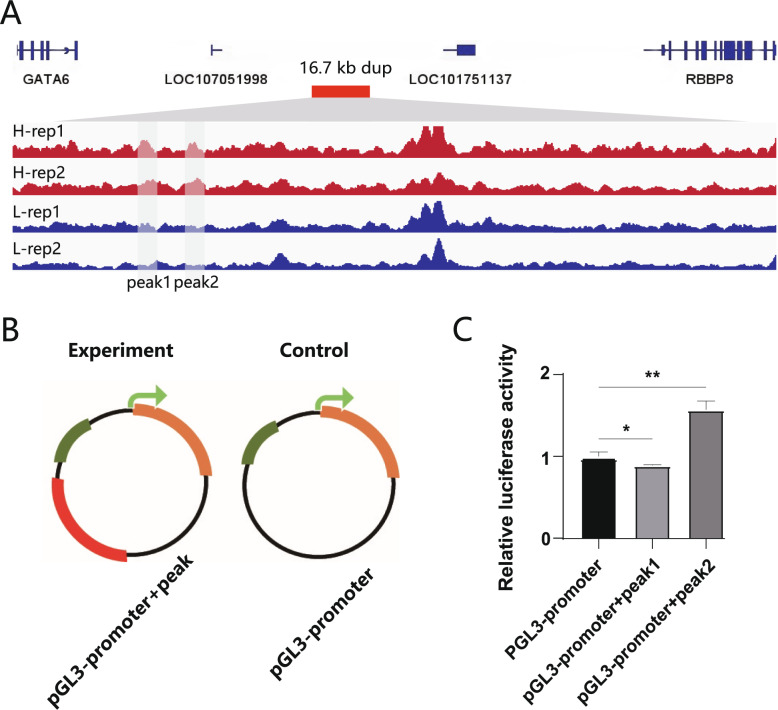
Screening and validating differential regulatory elements (DRE) in the duplication region. **A** ATAC-seq analysis for the identification of DRE. The red block represents the duplication on chromosome 2, while the gray transparent blocks represent DRE regions. H represents individuals with EWs greater than 40.2 g and multiple copies, and L represents individuals with EWs less than 33.5 g and single copy. **B** Construction diagram of double fluorescence experimental carrier. **C** Dual-luciferase assay validating the regulatory activity of peak1 and peak2, with ** indicating *P* < 0.01 significance, and * indicating *P* < 0.05 significance. Error line represents standard deviation (SD)

To further investigate the dup function, we analyzed ATAC-seq data from liver and oviduct ampulla tissues of high and low EW groups to identify differentially open chromatin regions (OCAs). We identified 125 OCAs (up: 29, down: 96) in the liver and 40 OCAs (up: 18, down: 22) in the oviduct ampulla (Supplementary Fig. 10). Of all the OCAs, only two OCAs were detected within the dup region in the liver (Fig. 5A). Dual-luciferase assays indicated that a 245 bp OCA (chr2: 102,725,259 ~ 102,725,504 bp) had enhancer activity in the liver (Fig. 5C), aligning with the expression trend of GATA6 in the liver. These findings suggest that the increased copy number of the dup may elevate GATA6 expression located 66.6 kb upstream.

## Discussion

DZH chickens represent a valuable genetic resource for understanding the genetic basis of EW. Based on quantitative trait inheritance theory [34], the significant EW differences observed between DZH chickens and other yellow-feathered breeds may be driven by causal mutations. Previous GWAS studies have revealed multiple chromosome candidate regions associated with EW in yellow-feathered chickens [35, 36], including two EW associated SVs on chromosome 2 [37, 38]. While, GWAS and SV-GWAS analyses in DZH and WCH chickens identified a new highly associated genomic region on chromosome 2 containing a duplication. PCR further validated this duplication in 456 yellow-feathered chickens and 118 F_2_ populations, where significant associations with EW were observed. This finding underscores the important role of the dup in regulating EW in yellow-feathered chickens.

Although the metabolic regulation of GATA6 in mammals has been extensively studied [39], its role in poultry reproductive traits has not been clearly defined. To our knowledge, this is the first study to demonstrate the possible role of GATA6 in regulating EW in yellow-feathered chickens. The 17.1 kbduplication located 66.6 kb downstream of GATA6 was significantly associated with EW. GATA6, a zinc-finger transcription factor, is integral to vertebrate development, particularly in the growth of vital organs such as the liver and heart [40, 41]. Related studies have also shown that in mice, GATA6 deletion leads to a failure of germ expansion and significant changes in genes related to lipid metabolism [41, 42]. As the primary site of yolk precursor synthesis (e.g., vitellogenin), the liver is regulated by estrogen and transports these compounds via the bloodstream to oocytes [43–46]. In chickens, liver development and aging are closely linked to lipid synthesis capacity, which increases as the liver matures and decreases as it ages [45–47]. Since lipid metabolic pathways greatly influence yolk lipid composition and quality [48–52], we speculated that GATA6 may directly affect EW by regulating lipid metabolity-related genes, or indirectly affect egg weight by influencing liver development.

Structural variants (SVs), which comprise a substantial portion of genetic variation among individuals, can significantly influence the expression of nearby genes [53–55]. In this study, LOC107051998 and LOC101751137 near the dup region were highly homologous to GATA6, suggesting they may be products of a GATA6 duplication event or members of the same gene family. It is plausible that these genes have similar functions or are regulated by the same variant [56, 57]. Based on our luciferase assay results, we identified an OCA with enhancer activity within the dup region. This finding suggests that the enhancer within the dup region may influence GATA6 expression, contributing to EW regulation.

## Conclusion

In conclusion, our results show that duplication located in a genomic region of chromosome 2 is highly correlated with the EW of yellow-feathered chickens. A 245 bp Enhancer Identified within the duplication may affect EW mediated by GATA6. The identification of a 17.1 kb tandem duplication provides an important theoretical basis for the genetic selection of high EW lines.

## Supplementary Information

Supplementary Material 1.

## Data Availability

The fastq data of WCH chickens have been deposited in the Genome Sequence Archive (GSA) under BioProject accession no PRJCA029895, PRJCA025339 and PRJCA032111.

## Abbreviations

CNVs: Copy number variations
CTSD: cathepsin D
DEGs: Differentially expressed genes
DZH: Danzhou
EW: Egg weight
GWAS: Genome-wide association study
HBM: Huaibei Mottled
HL: Huanglang
IGV: Integrative Genomics Viewer
LD: Linkage disequilibrium
MAF: Minor allele frequency
NDY: Ningdu Yellow
PCA: Principal component analysis
QTL: Quantitative trait loci
Rbp4: Retinol-binding protein 4
RJ: Red gallus
SNJL: Shennongjia Large
SNPs: Single nucleotide polymorphisms
SVs: Structural variations
VLDLR: Very low-density lipoprotein receptor
WCH: Wenchang
WGS: Whole-genome sequencing
WH: Wuhua
YW: Yolk weight
YYL: Yunyang Large
ZYTY: Zhengyang Three-Yellow
