# Supplementary material for: A 17.1 kb duplication downstream GATA6 is strongly associated with egg weight in chicken
Source: BMC Genomics. 2025 Aug 20;26:765. doi: 10.1186/s12864-025-11888-0 (PMC12366128; doi:10.1186/s12864-025-11888-0)
Supplement: Supplementary file 1 — Supplementary Material 1. [file 12864_2025_11888_MOESM1_ESM.docx]

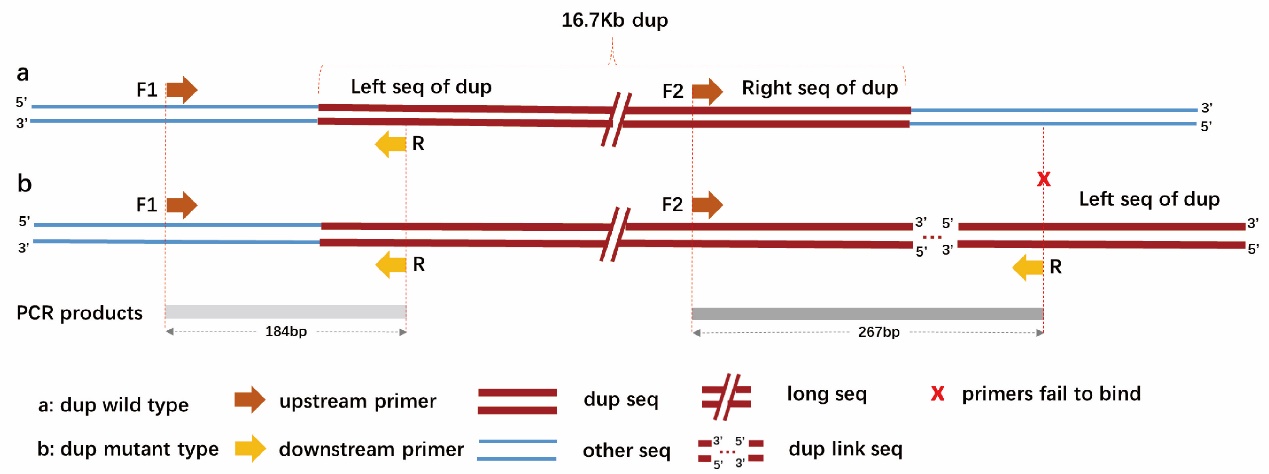


**Supplementary figure 1.** Design principles of dup PCR primers. a indicates a single-copy individual, and b indicates a multi-copy individual.


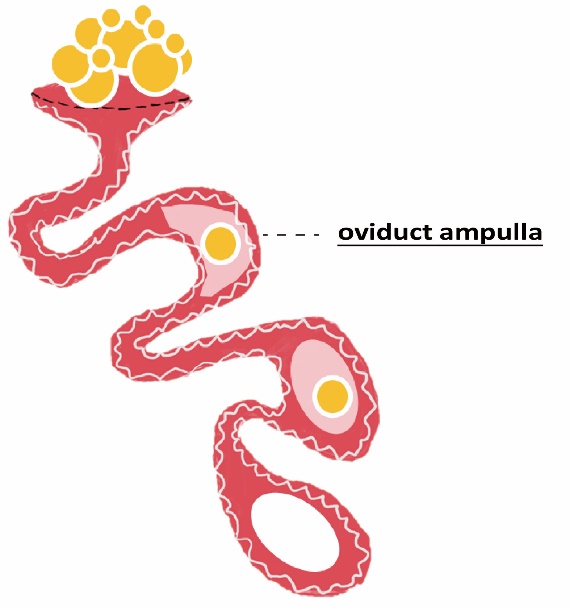


**Supplementary figure 2.** Tissue sampling diagram.


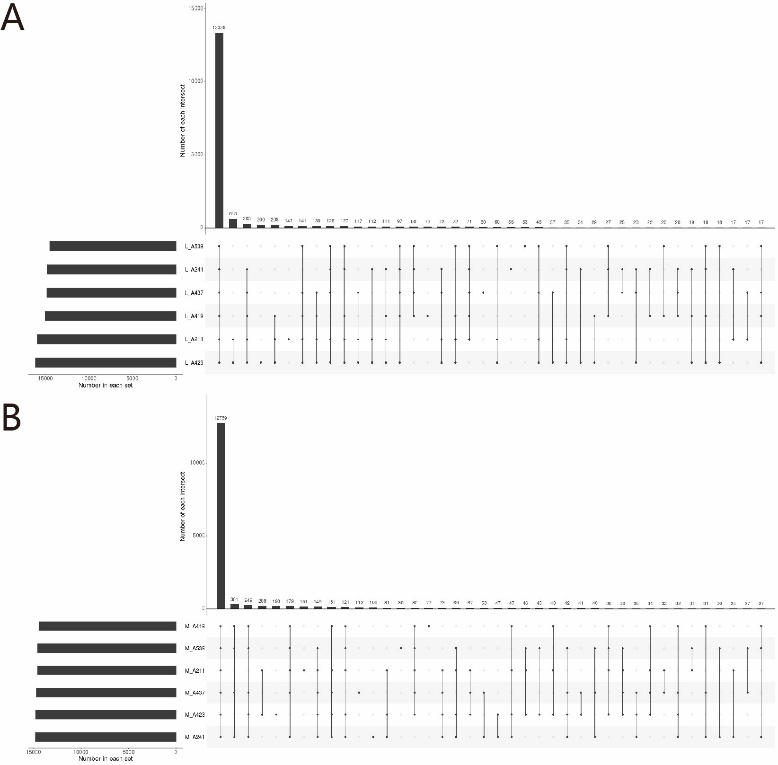


**Supplementary figure 3. UpSet plot of the distribution of different genes in multiple samples.** (A) liver; (B) oviduct ampulla.


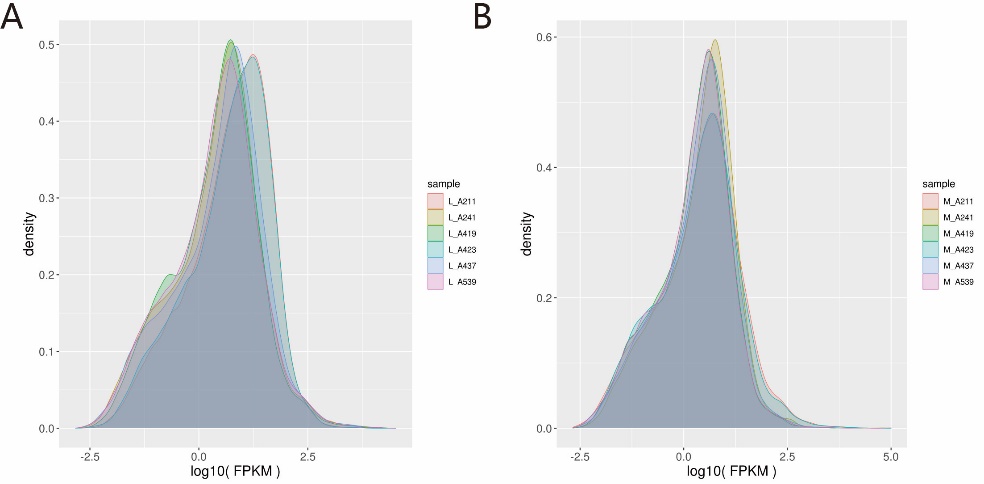


**Supplementary figure 4. Density plot of FPKM.** (A) liver; (B) oviduct ampulla.


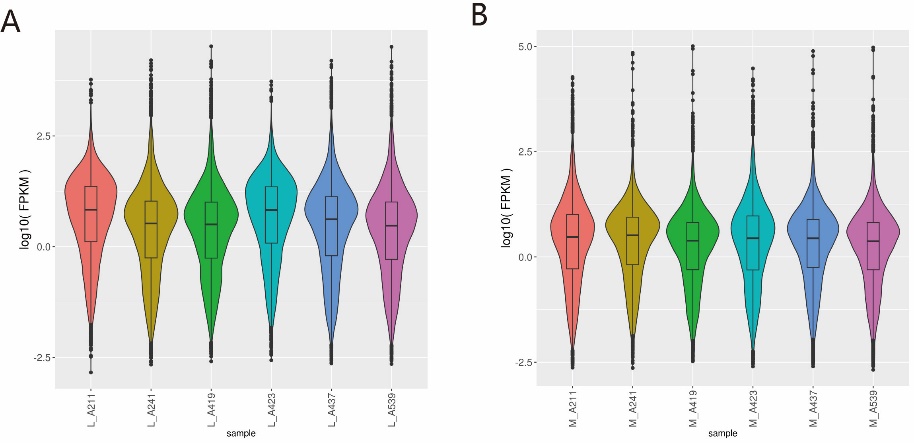


**Supplementary figure 5. Violin plot of FPKM.** (A) liver; (B) oviduct ampulla.


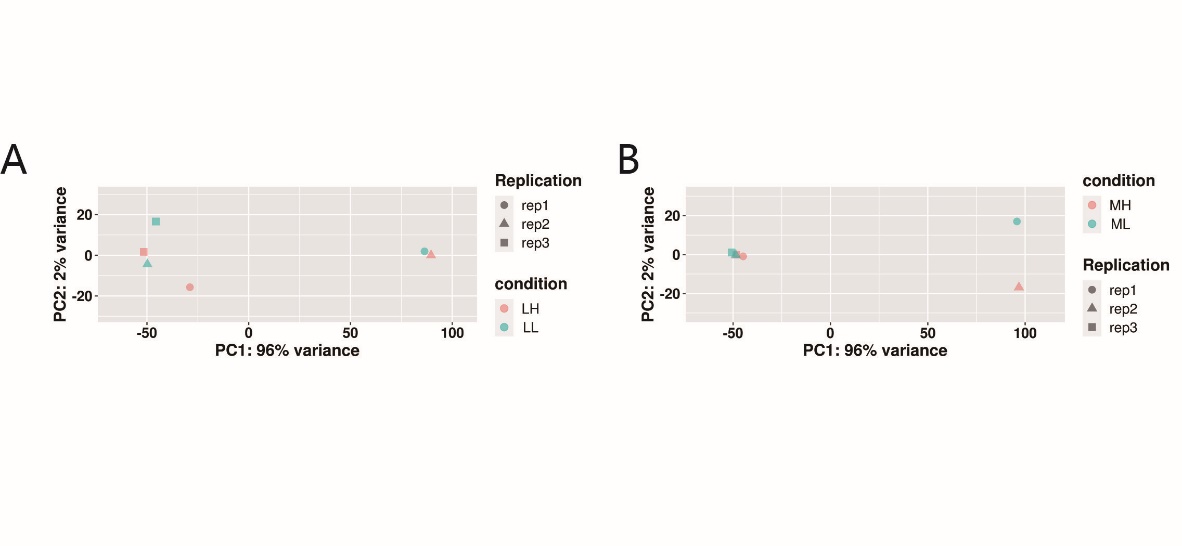


**Supplementary figure 6.** PCA map of RNA-seq data. (A) liver; (B) oviduct ampulla.


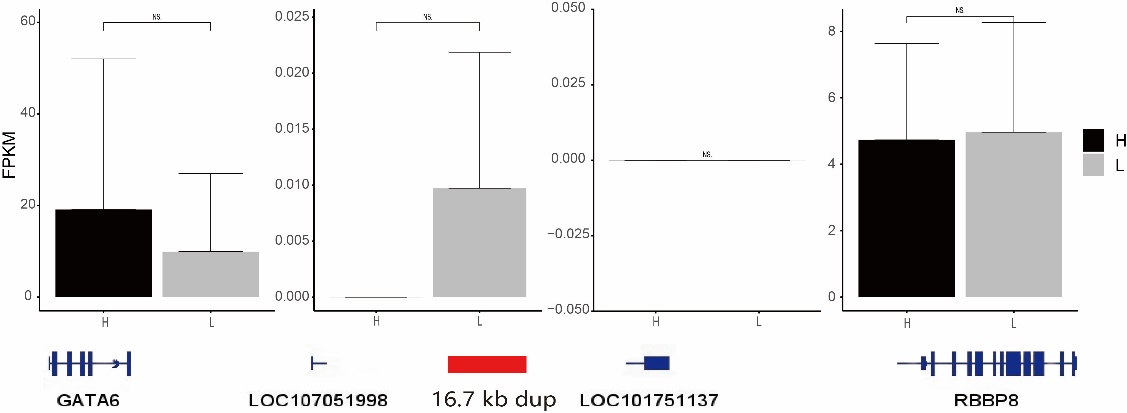


**Supplementary figure 7.** ARGs expression in oviduct ampulla.


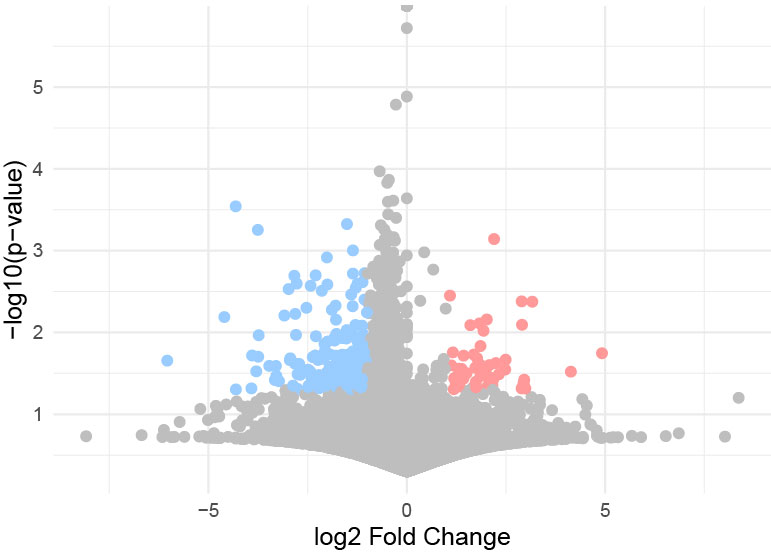


**Supplementary figure 8.** DEGs volcano map of oviduct ampulla


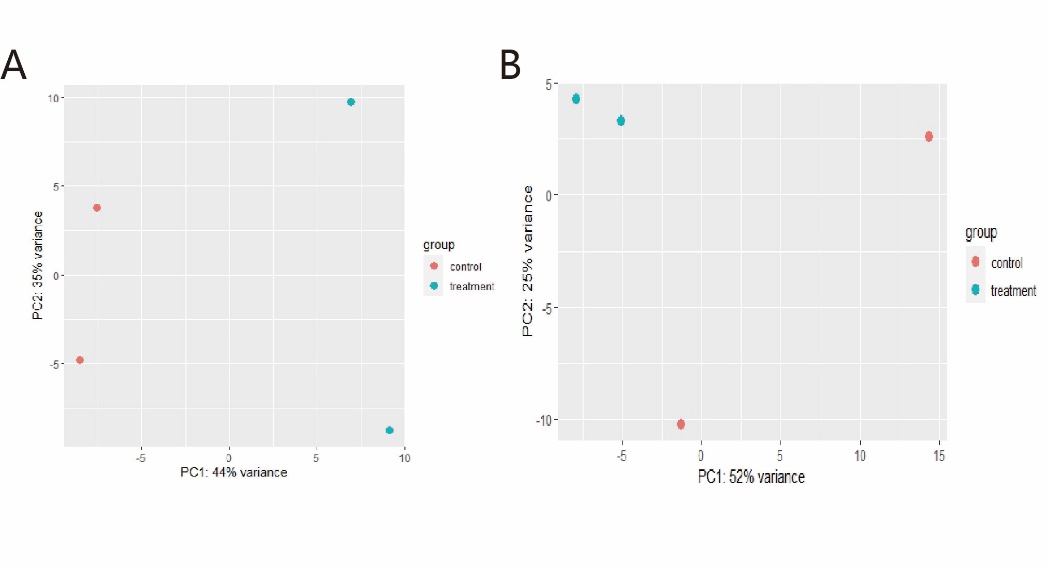


**Supplementary figure 9.** PCA map of ATAC-seq data. (A) liver; (B) oviduct ampulla.


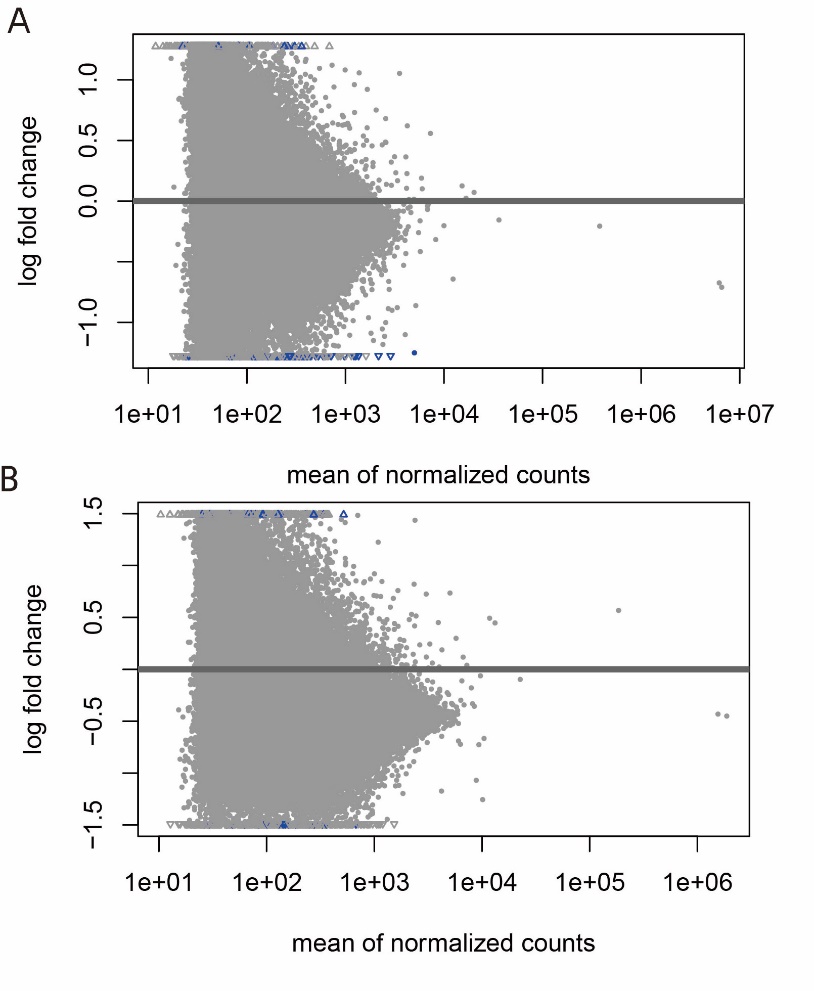


**Supplementary figure 10.** Differential open chromatin areas. (A) liver; (B) oviduct ampulla.


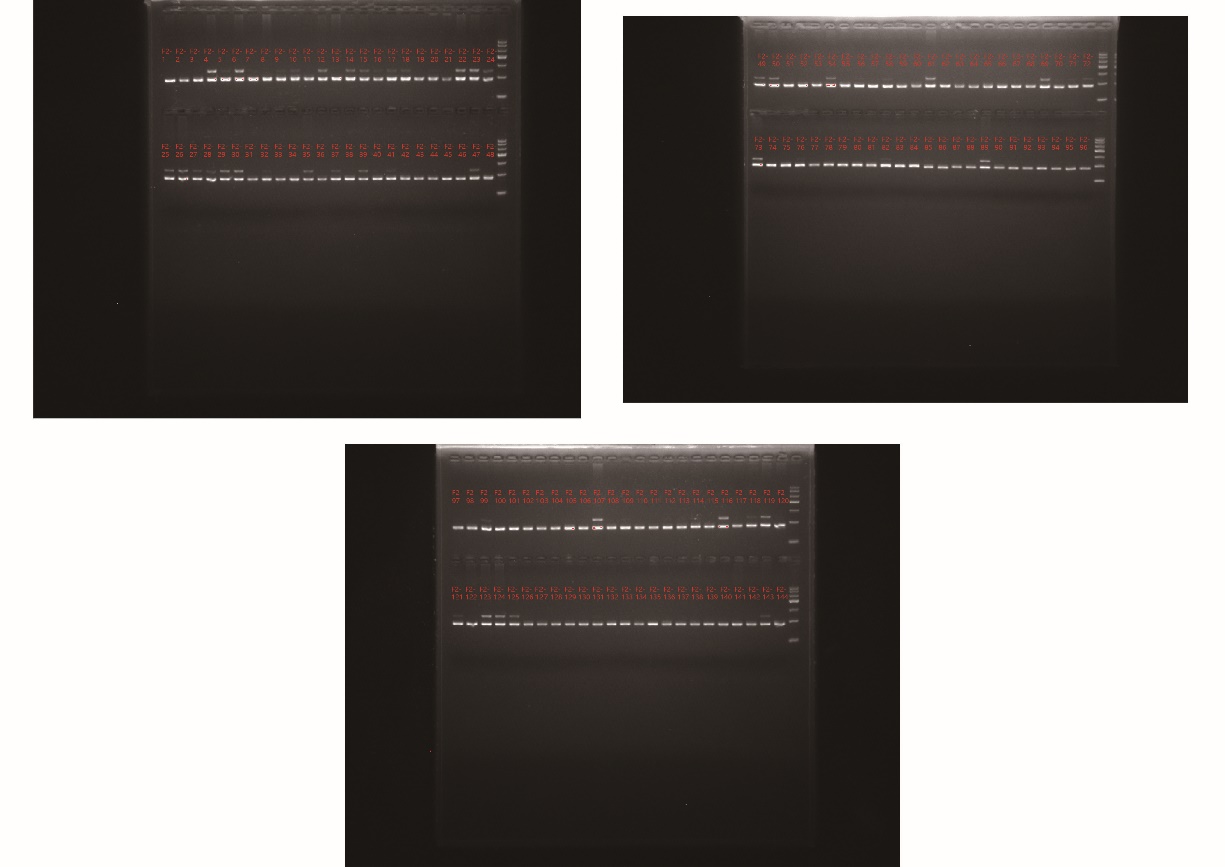


**Supplementary figure 11.** F2 population gel/blot images.


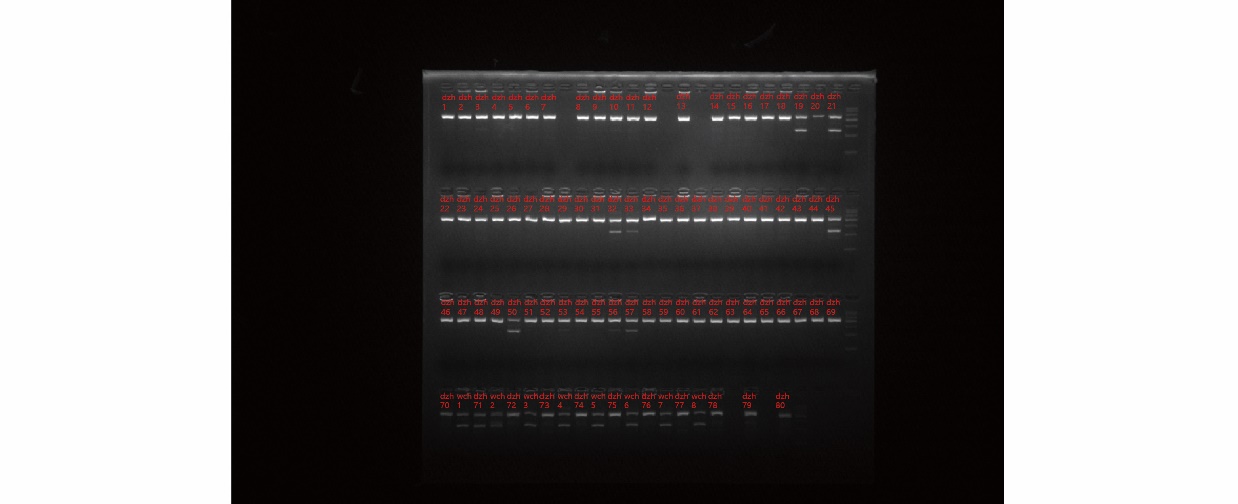


**Supplementary figure 12.** F0 population gel/blot images. dzh stands for maternal Danzhou chickens and wch stands for paternal Wenchang chickens


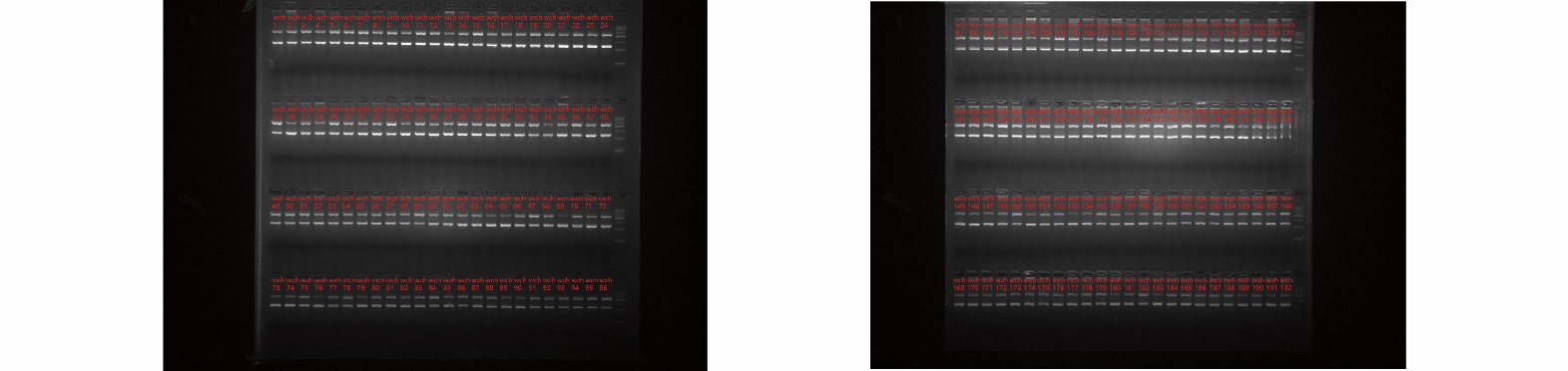


**Supplementary figure 13.** F2 population gel/blot images. wch stands for Wenchang chickens


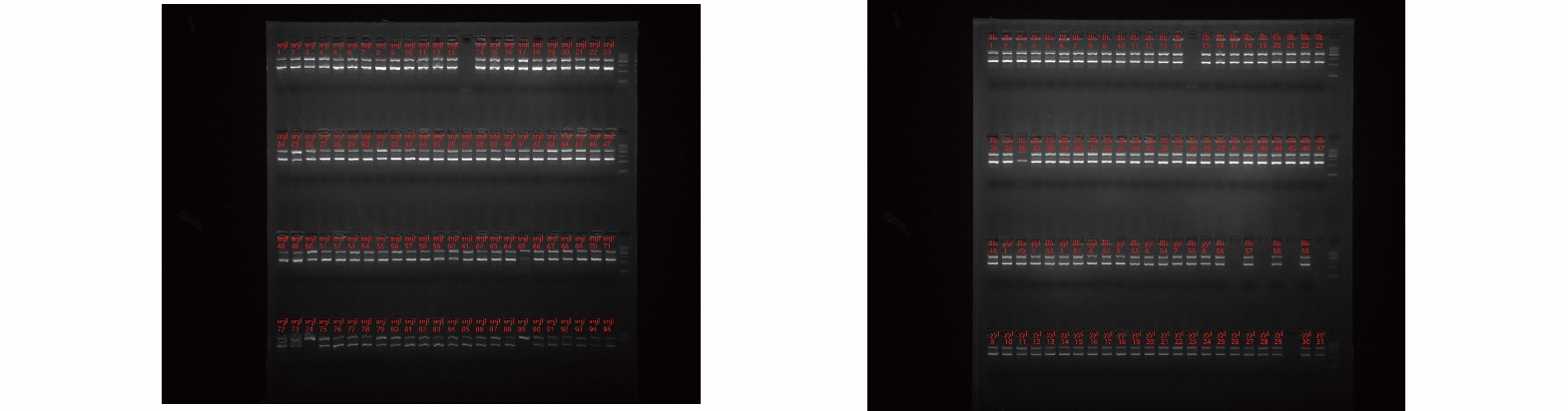


**Supplementary figure 14.** F2 population gel/blot images. snjl stands for Shennongjia Large Chicken, llb stands for Luling Black Chicken and yyl stands for Yunyang Large Chicken.
